# Recurrence in isolated distal DVT after anticoagulation: a systematic review and meta-analysis of axial and muscular venous thrombosis

**DOI:** 10.1186/s12959-024-00623-6

**Published:** 2024-07-01

**Authors:** Wen-Tao Yang, Zhen-Yi Jin, Chun-Min Li, Jia-Hao Wen, Hua-Liang Ren

**Affiliations:** Department of Vascular Surgery, Beijing Chaoyang Hospital, Capital Medical University, No.8 Gongti South Road, Beijing, 100020 China

**Keywords:** Deep venous thrombosis, Anticoagulants, Pulmonary embolism, Venous thromboembolism, Recurrence

## Abstract

**Objective:**

To identify recurrent venous thromboembolism (VTE) after discontinuation of anticoagulation in patients with isolated distal deep vein thrombosis based on its anatomic localization (axial or muscular veins).

**Methods:**

Data were sourced from PubMed, Embase, Cochrane Library, Web of Science, and ClinicalTrials.gov databases in the time period up to October 2023. The study followed PRISMA guidelines using a registered protocol (CRD42023443029). Studies reporting recurrent VTE in patients with axial or muscular DVT were included in the analysis.

**Results:**

Five studies with a total of 1,403 participants were evaluated. The results showed a pooled odds ratio of 1.12 (95% confidence interval 0.77–1.63) between axial and muscular DVT. Heterogeneity was low (I^2^ = 0%, p = 0.91) and there was no significant difference in the rate of recurrent VTE between axial and muscular DVT in each subgroup.

**Conclusions:**

Muscular and axial DVT showed comparable recurrent VTE rates after anticoagulation. However, uncertainties regarding the possibility of recurrence affecting the popliteal vein or resulting in pulmonary embolism following muscular DVT anticoagulation persisted. Randomized trials in patients with isolated distal DVT are still needed to clarify its prognosis for different anatomical thrombus locations.

**Supplementary Information:**

The online version contains supplementary material available at 10.1186/s12959-024-00623-6.

## Introduction

Isolated distal deep vein thrombosis (IDDVT) accounts for 30–56% of all DVT cases [1–3]. The rate of proximal extension or pulmonary embolism (PE) in patients with untreated IDDVT can be as high as 22% [4].

**Fig. 1:**
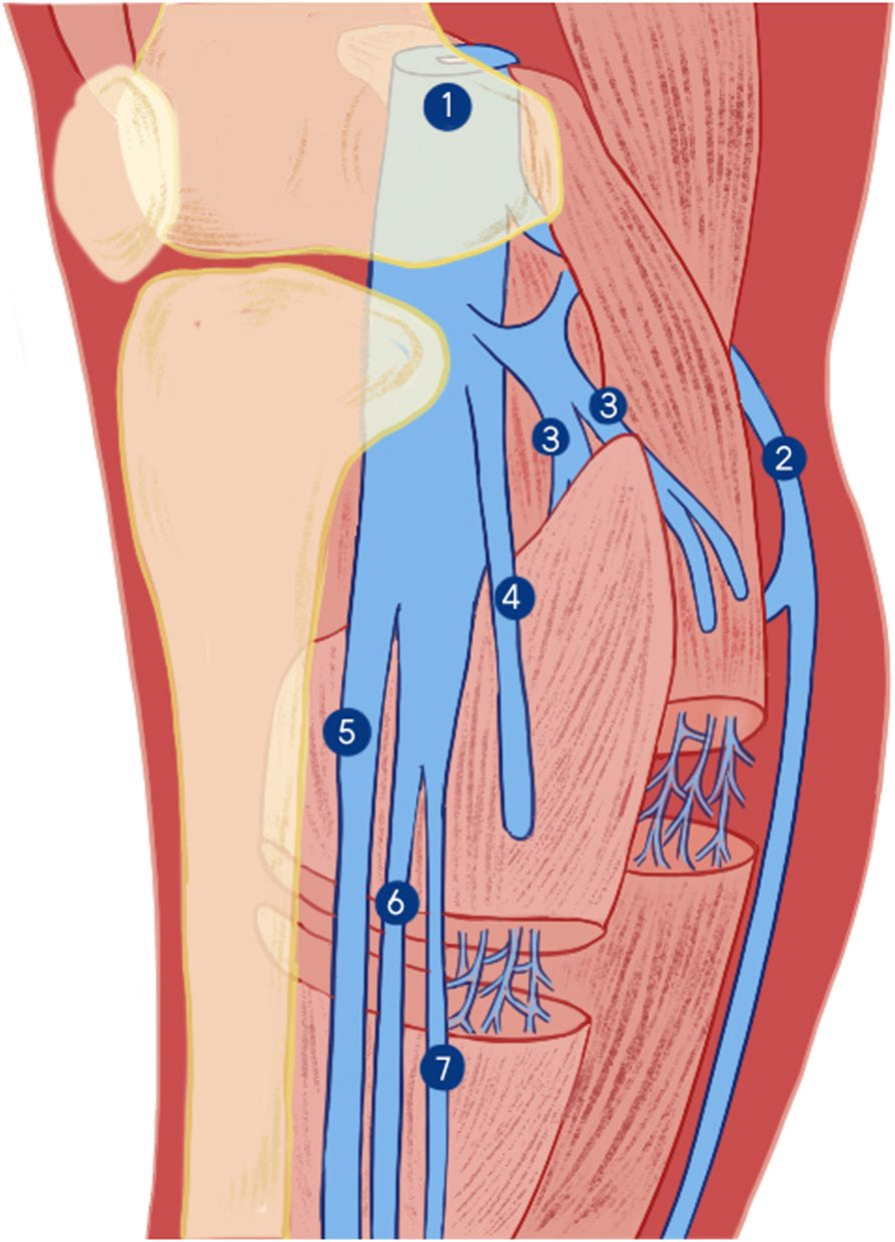
Anatomical diagram of calf veins. 1.Popliteal vein; 2.Short saphenous vein; 3.Gastrocnemius veins; 4.Soleus vein; 5.Anterior tibial vein; 6.Peroneal vein; 7.Posterior tibial vein

IDDVT involves either the axial (posterior tibial, peroneal, and anterior tibial) or muscular (soleal and gastrocnemius) deep veins of the calf. Deep muscle veins are uniquely classified and run as sinusoids within the corresponding muscles of the same name. Soleal sinusoids may drain into the midperoneal or posterior tibial veins, whereas gastrocnemius sinusoids may empty directly into the popliteal vein. These differences lead to muscular vein reduced diameter and length, resulting in smaller thrombosis volumes and greater distance from the proximal veins than the axial deep veins (Fig. 1) [1, 5, 6]. Therefore, there may be differences in the progression of these two thrombus types.

The optimal management approach for IDDVT is the subject of ongoing debate, as reflected by heterogenous guideline recommendations [7–9]. Compared with continuous deep vein imaging suggeated by the American College of Chest Physicians, the European Society of Cardiology suggests that individuals at low risk of recurrence should be anticoagulated with LMWH for a shorter period (4–6 weeks), but lower anticoagulant doses, or ultrasound surveillance may be considered. There is currently no agreement on whether to categorize muscular DVT (MDVT) separately in the treatment of IDDVT. Some studies have reported the venous thrombosis propagation rate of MDVT without anticoagulation treatment. Retrospective studies by Sales et al. [10], Lautz et al. [11], and Kret et al. [12] revealed venous thrombosis propagation rates without anticoagulation treatment to be 28%, 30%, and 25%, respectively. Prospective studies by MacDonald et al. [13] and Schwarz et al. [14] reported venous thrombosis propagation rates of 16% and 25%, respectively. However, the findings by McDonald et al. demonstrated that only 2.9% of all MDVT cases progressed to the popliteal vein level. In another randomized controlled study conducted by Schwarz et al. [15], the rate of thrombus extension in MDVT without anticoagulation was 4%. This rate was influenced by the inclusion of low-risk individuals in the study cohort. In addition, international guidelines often provide recommendations with low­to­moderate certainty, reflecting the lack of robust clinical trial evidence. Studies from routine clinical practice have indicated that most patients with IDDVT are treated with anticoagulants [2, 16–18]. In the present study, a meta-analysis of randomized and cohort studies was performed in patients with IDDVT to assess whether there was a difference in the occurrence of recurrent VTE after anticoagulation treatment between MDVT and axial DVT (ADVT) and to explore the differences in progression of the two thrombus types.

## Methods

The Preferred Reporting Items for Systematic Reviews and Meta-Analysis (PRISMA) guidelines were followed [19]. The study protocol was pre-registered on PROSPERO (CRD42023443029).

### Search strategy

A systematic search was performed using electronic databases (PubMed, Embase, Cochrane Library, and Web of Science) up to October 2023 to identify all available studies on anticoagulation for IDDVT classified as ADVT and MDVT. ClinicalTrials.gov was also searched to identify any ongoing randomized controlled trials (RCTs). Appropriate medical subject heading terms and free word searches were utilized. The full search strategy is available in Supplementary Material (Table S1). References for the included studies were screened for further eligible articles. No language restriction was used.

### Study selection

Two independent authors (WTY and ZYJ) analyzed the lists of retrieved articles and performed the study selection. Any disputes were resolved by a third reviewer (HLR).

The included articles had the following characteristics: (i) RCTs or prospective observational studies; (ii) objective diagnosis by ultrasonography (US) or venography of ADVT (i.e., posterior tibial, peroneal, and anterior tibial vein) and MDVT (soleal and gastrocnemius vein); (iii) intervention (anticoagulation for at least four weeks); (iv) availability of data on the incidence of DVT recurrence, proximal propagation, and/or PE; and (v) a minimum of 50 patients with ADVT or MDVT. Excluded articles had the following characteristics: (i) retrospective studies; (ii) < 3 months of follow-up; (iii) patients aged < 18 years; (iv) DVTs with proximal extension into popliteal, femoral, and oriliac segments; (v) endovascular or surgical interventions (e.g., catheter-directed thrombolysis); and (vi) case series, case reports, review articles, letters, and editorials.

The screening process was conducted using EndNote X9 (Clarivate, Chandler, AZ, USA). All titles and abstracts retrieved from the initial search were screened. Articles meeting the inclusion criteria were subjected to an independent full-text review for final eligibility and data extraction. Relevant articles with incomplete information based on the title or abstract were also subjected to a full-text evaluation.

### Data extraction

All original articles selected for inclusion in the meta-analysis were reviewed and the following data were extracted when available: general data (year of publication, design), population characteristics (number and type of included patients), and method used for IDDVT diagnosis, treatment (types of anticoagulant, dose, and duration), and follow-up (duration, surveillance method). For patients with IDDVT who underwent anticoagulation, information on the following outcomes were collected: recurrent DVT, proximal extension of IDDVT, and PE. Two reviewers (WTY and ZYJ) extracted the data independently using a template in Microsoft Excel. If the pre-specified data elements were not found during the review of published trial results, the authors of the publications were contacted to obtain additional study-level summary information. Investigators from one trial [20] provided additional data upon request.

### Quality assessment

Quality assessment was performed independently by two reviewers (WTY and ZYJ) using the revised tool for risk of bias in randomized trials (RoB 2 tool). Prospective cohort study quality was assessed using the Newcastle–Ottawa Quality Assessment Scale (NOS). Any discrepancies were mediated by a third reviewer (HLR). The Grading of Recommendations, Assessment, Development, and Evaluation (GRADE) profiler tool was then used to assess the reporting quality of major study outcomes [21].

### Outcomes

The primary outcome of our meta-analysis was recurrent VTE defined as the composite of progression of IDDVT, recurrent IDDVT, proximal DVT, and PE occurring during the study period. Progression of IDDVT was defined as a compression ultrasonography confirmed extension of isolated distal DVT to the calf trifurcation (if previously not involved), popliteal, femoral, or iliac vein using a standardised compression ultrasonography protocol. Recurrent IDDVT was defined as a new distal DVT in the contralateral leg, lack of compressibility of a previously compressible vein in the ipsilateral leg, or an increase of at least 2 mm in the diameter of the residual thrombus during compression in a previously non-compressible vein. Proximal DVT was defined by a new proximal DVT in the contralateral leg. US or venography were accepted for confirmation of recurrent VTE and a computed tomography scan or ventilation–perfusion scan for PE. In addition, subgroup analyses were performed based on the study type, with population excluding active cancer or previous VTE, and follow-up time.

### Statistical analysis

The odds ratio (OR) and 95% confidence interval (CI) were calculated for each study. The results were compared using a fixed-effects model [22]. Cochran’s χ^2^ test and I^2^ test for heterogeneity were used to assess between-study heterogeneity [23]. Statistically significant heterogeneity was considered to be present at P < 0.10 and I^2^ > 50%. Analyses were performed with Review Manager 5.4 (The Cochrane Collaboration, Oxford, UK) and Stata version 17 (StataCorp LP, College Station, TX, USA).

## Results

**Fig. 2:**
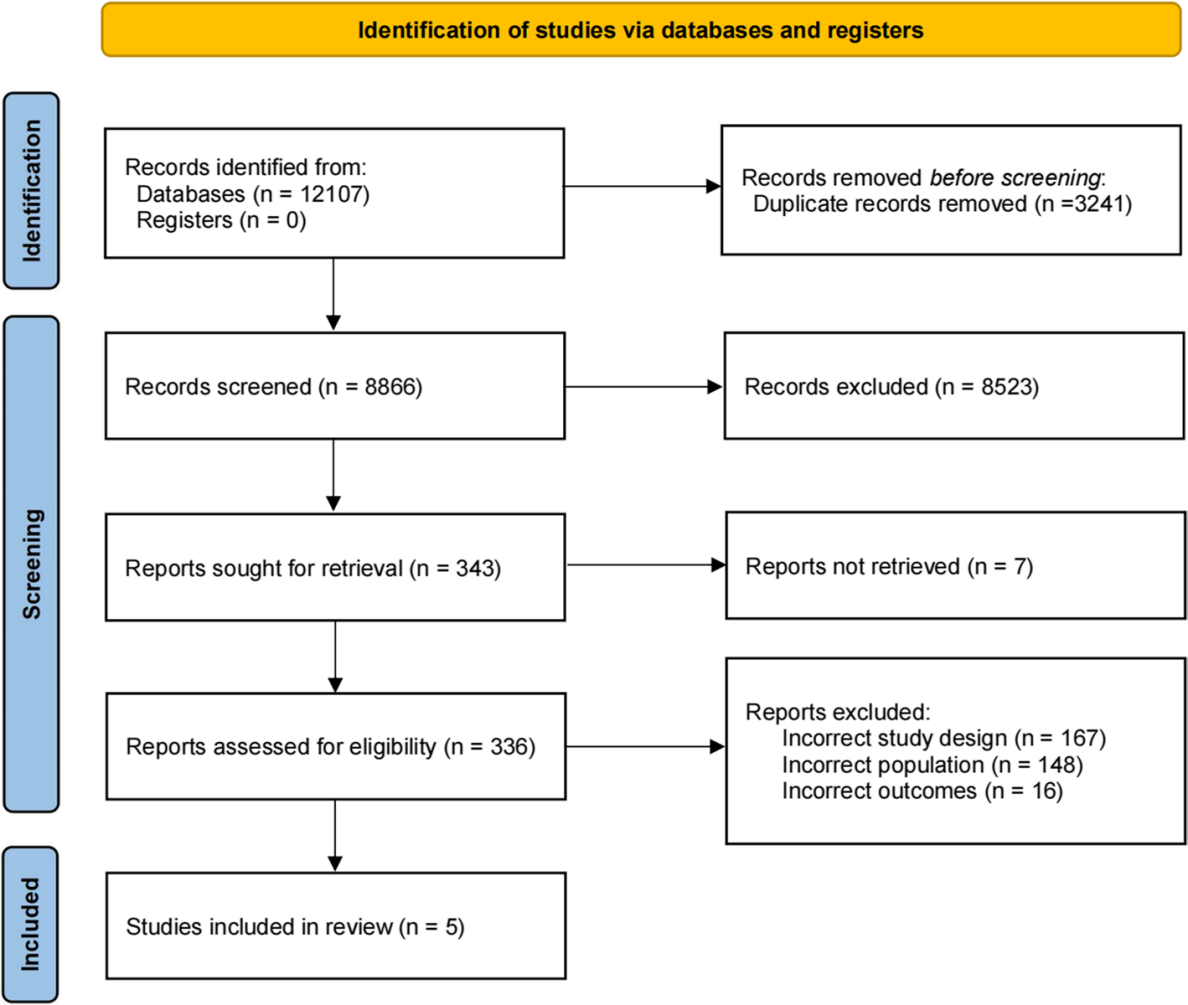
Preferred reporting items for systematic reviews and meta-analyses (PRISMA) flow diagram for studies reporting the incidence of recurrent events after isolated distal deep vein thrombosis at different anatomical locations included in the present meta-analysis

Search results are represented in the PRISMA diagram (Fig. 2).

### Study characteristics

The incidence of recurrent VTE after ADVT and MDVT was analyzed based on two RCTs and three prospective cohort trials. The studies were conducted in Italy [24, 25], France [26], Norway [27], and Canada, France, and Switzerland [20] and published between 2012 and 2023. They featured 90–475 participants, with 1,403 patients included overall across the five studies. Most studies used compression US for initial diagnosis of IDDVT. Four studies followed up patients within three years after the first DVT, and one study followed up patients for an average of 4.7 years. In terms of intervention, almost all patients received anticoagulantion therapy and there were variations among the studies regarding the specific medications used and treatment duration. Further details on characteristics of each study are available in Table 1.

**Table 1:** Characteristics of eligible studies

Study	Country	Design	Diagnosis based on	Inclusion criteria	Intervention	Extracted IDDVT anatomical location^*^	Primary outcome / end point	Length of follow up	Rates of recurrent VTE
Ageno 2022 [24]	Italy	Randomized, double blind, placebo controlled clinical trial	CUS	Adults with objectively diagnosed symptomatic IDDVT of the legs	Rivaroxaban 15 mg twice daily for 3 weeks followed by rivaroxaban 20 mg once daily for 3 weeks. At the end of the 6 weeks, patients who had not developed thrombotic or haemorrhagic complications were randomised to receive either rivaroxaban 20 mg or placebo once daily for an additional 6 weeks	140 Axial 262 Muscle	Composite of progression of IDDVT, recurrent IDDVT, proximal DVT, symptomatic PE or fatal PE	24 months	Axial 15.0% Muscle 15.6%
Galanaud 2014 [26]	France	Prospective, multicenter study	CUS	Patients aged ≥ 18 years whose index VTE event was a first objectively confirmed symptomatic IDDVT or IPDVT	Anticoagulation Median of 92 days (IQR 61–123)	100 Axial 212 Muscle	First symptomatic VTE recurrence after stopping anticoagulants	900 days	Axial 4.0% Muscle 4.2%
Jørgensen 2023 [27]	Norway	Prospective, single center study	CUS	Patients aged ≥ 18 years with objectively verified first-time IDDVT, only the symptomatic leg was imaged	LMWH 49 (10.3%) VKA 168 (35.4%) DOAC 258 (54.3%) Median of 92 days (IQR 91–114)	385 Axial 90 Muscle	Symptomatic DVT or PE	Mean of 4.7 years (maximum of 14.9 years)	Axial 15.1% Muscle 12.2%
Righini 2016 [20]	Canada, France, and Switzerland	Randomized, double-blind, placebo-controlled trial	CUS	Outpatients with a first, acute, symptomatic, objectively confirmed calf DVT	Nadroparin 171 UI/kg vs. placebo for 42 days All patients used graduated elastic compression stockings (30 mm Hg) daily	61 Axial 63 Muscle	Composite endpoint of extension of calf DVT to proximal veins, contralateral proximal DVT, or symptomatic PE	90 days	Axial 3.3% Muscle 1.6%
Sartori 2014 [25]	Italy	Prospective, single center study	D-dimer testing and CUS	Symptomatic outpatients with IDDVT	2 no anticoagulation 56 LMWH for 1 month 32 VKA for 3 months All patients were instructed to wear 30–40 mmHg graduated compression knee high elastic stockings	41 Axial 49 Muscle	Composite of PE, proximal DVT, IDDVT recurrence / extension, cardiovascular death	Median ± SD: 730 ± 295 days	Axial 22.0% Muscle 16.3%

*DVT* Deep vein thrombosis, *CUS* Compression ultrasonography, *IDDVT* Isolated distal DVT, *PE* Pulmonary embolism, *VTE* Venous thromboembolism, *IPDVT* Isolated proximal DVT, *LMWH* Low-molecular-weight heparins, *VKA* Vitamin K antagonists, *US* Ultrasonography, *ICVT* Isolated calf vein thrombosis

### Risk of bias assessment and quality assessment of outcomes

Two RCTs were appraised using the RoB 2 tool and three prospective cohort studies were appraised using the NOS. Overall, the risk of bias was low in all studies. A summary of the quality assessment is provided in the Supplementary Material (Table S2). However, the majority of the extracted data in RCTs were based on subgroup analyses, thereby raising the potential risk of bias due to the variation in baseline characteristics between groups. Furthermore, the included studies encompassed observational studies. Thus, the quality of evidence was graded as low according to the GRADE criteria. A funnel plot was not generated to assess the publication bias since fewer than 10 studies were included in the analysis [28].

### Recurrence in two types of IDDVT

**Fig. 3:**
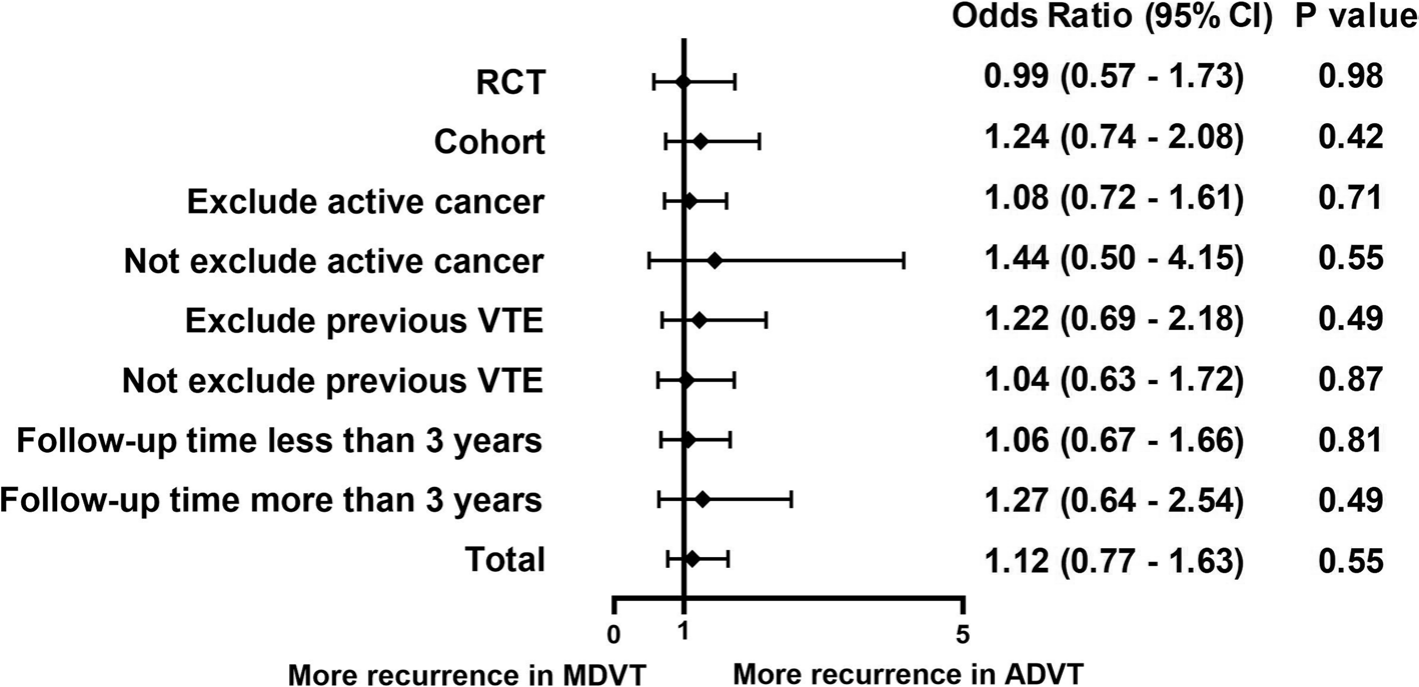
Recurrent venous thromboembolism (VTE) in patients with axial deep venous thrombosis (ADVT) or muscular deep venous thrombosis (MDVT)

The recurrent rates of both types of IDDVT during the follow-up period of each included study are shown in Table 1. Data from patients with two types of IDDVT who experienced recurrent VTE were weighted and pooled using fixed-effects meta-analysis. Comprehensive analysis of these studies revealed the same rate of recurrent VTE in ADVT compared to that in MDVT (FE model: OR, 1.12; 95% CI, 0.77–1.63, I^2^ = 0%, p = 0.91). There was no significant difference in the rate of recurrent VTE between ADVT and MDVT in each subgroup (RCT, FE model: OR, 0.99; 95% CI, 0.57–1.73, I^2^ = 0%; cohort, FE model: OR, 1.24; 95% CI, 0.74–2.08, I^2^ = 0%; excluding active cancer, FE model: OR, 1.08; 95% CI, 0.72–1.61, I^2^ = 0%; not excluding active cancer, FE model: OR, 1.44; 95% CI, 0.50–4.15, I^2^ = 0%; excluding previous VTE, FE model: OR, 1.22; 95% CI, 0.69–2.18, I^2^ = 0%; not excluding previous VTE, FE model: OR, 1.04; 95% CI, 0.63–1.72, I^2^ = 0%; follow-up time less than three years, FE model: OR, 1.06; 95% CI, 0.67–1.66, I^2^ = 0%; follow-up time more than three years, FE model: OR, 1.27; 95% CI, 0.64–2.54, I^2^ = 0%; Fig. 3, details are shown in Figure S1).

## Discussion

The present meta-analysis examined the rate of recurrent VTE in patients with ADVT compared to MDVT after anticoagulation. The main study finding was that the rate of recurrence was similar in both types of IDDVT after anticoagulation within approximately five years. This evidence implies a potential similarity in the progression of ADVT and MDVT. The anatomical differences in physiology of axial and muscular veins did not appear to affect the rate of recurrent VTE in patients with IDDVT.

International guidelines favor serial imaging rather than anticoagulation in isolated MDVT [8]. However, the rate of recurrence in a retrospective study by Ho et al. [29] was similar between intramuscular IDDVT and non muscular IDDVT (14% vs. 7%, p = 0.13) and despite the lower rate of major VTE (defined as above-knee or proximal DVT and PE) recurrence in the intramuscular IDDVT population. In another retrospective study by Utter et al. [30], therapeutic anticoagulation did not show a significant association with proximal DVT or PE in patients with ADVT (adjusted OR, 0.52; 95% CI, 0.14–1.90). However, a notable association was observed among patients with MDVT (adjusted OR, 0.12; 95% CI, 0.03–0.53). Some retrospective studies have presented conflicting conclusions based on this study. Kuczmik et al. [31] compared the difference in recurrent VTE between ADVT and MDVT after anticoagulation. This group noted that recurrent VTE was three times more common in ADVT compared to MDVT, particularly if anticoagulants were withheld. In general, these studies tend to indicate that MDVT is a more benign condition compared to ADVT. However, it is worth noting that in the study by Kuczmik et al. [31], the ADVT group included patients with both ADVT and MDVT. In the study by Galanaud et al. [26], which categorized IDDVT into the ADVT, MDVT, and ADVT + MDVT groups, the 900-day cumulative recurrence rates for the ADVT and MDVT groups were similar. However, the ADVT + MDVT group exhibited a higher cumulative recurrence rate. The possibility of a higher recurrence rate among patients with both types of thrombosis suggests that it may be more appropriate to analyze such patients in a separate group to obtain more accurate findings.

Regarding the rates of recurrent VTE, the studies included in our analysis demonstrate similarities to those related to MDVT or ADVT. According to Schwarz et al. [15], the rate of venous thrombosis propagation in MDVT patients treated with anticoagulation and compression stockings was 4% during the 3 month follow-up period. In their retrospective study on MDVT patients receiving anticoagulation treatment, Lautz et al. [11] indicated a venous thrombosis propagation rate of 12% during the follow-up period of up to 18.5 months. Furthermore, two RCTs by Horner et al. [32] and Righini et al. [20] compared the outcomes in patients with IDDVT who received anticoagulation treatment with those who did not. Both trials had a follow-up period of 3 months, and the rate of recurrent VTE was 11.4% and 6.2%, respectively.

Longer-term outcomes remain inconclusive. In the study conducted by Jørgensen et al. [27], the cumulative incidence of recurrent VTE after anticoagulation for ADVT and MDVT after ten years of follow-up was 29.1% (95% CI 17.5–40.2%) and 12.0% (95% CI 5.9–18.0%), respectively [27]. This suggested the possibility of disparate long-term outcomes between the two types of thrombosis. Nevertheless, this study reported an HR of 1.69 (95% CI 0.73–3.93) for ADVT when using MDVT as the reference group.

The management of IDDVT is currently undergoing continuous improvement. Present guidelines recommend that patients lacking severe symptoms or extension risk factors should undergo a two-week period of continuous deep vein imaging over anticoagulation treatment. However, the strength of the available evidence remains inadequate. Within this subset of IDDVT, the management of MDVT remains the subject of ongoing debate. Although continuous compression US is a viable choice for isolated MDVT, the majority of patients in clinical practice still opt for anticoagulantion therapy. This, in turn, complicates the execution of pertinent research. Therefore, the need for clinical RCTs focused on MDVT remains significant. Despite the present study indicating a similar recurrence rate between MDVT and ADVT after anticoagulation, distinctions in the occurrence of popliteal vein level involvement and PE within MDVT recurrence events remain unclear. Similarly, there is a paucity of research concerning recanalization and post-thrombotic syndrome rates for both thrombosis types following anticoagulation therapy.

The strengths of our study include comprehensive inclusion criteria and a thorough statistical analysis that yielded a credible result. Only prospective studies were included due to the serious risk of selective reporting that would be expected with the inclusion of retrospective studies. All of the included studies were published after 2010, reducing the heterogeneity caused by obsolete diagnostic techniques and treatment modalities. In addition, our meta-analysis also had some specific limitations. The majority of studies did not categorize the study population into distinct groups of MDVT and ADVT, resulting in most of the collected data being derived from subgroups. Populations also differed between studies. Due to lack of sufficient data, this study did not analyze individual outcomes like PE incidence with recurrence or proximal deep vein extension separately. Furthermore, the distribution of ADVT and MDVT was different in the included studies and the follow-up duration was inconsistent. While all participants received anticoagulation therapy, there was variability in anticoagulation type and duration.

## Conclusion

The post-anticoagulation recurrence rate for MDVT is comparable to that for ADVT. However, uncertainties persist regarding the likelihood of recurrence affecting the popliteal vein or resulting in PE following MDVT anticoagulation. Currently, there are few prospective studies dedicated exclusively to ADVT and MDVT. Instead, most data stem from subgroup analyses within broader investigations. In the future, the possibility of initiating clinical trials specifically targeting these two discrete thrombosis types could be considered in order to bridge these knowledge gaps.

### Supplementary Information

Supplementary Material 1.

Supplementary Material 2.

Supplementary Material 3.

## Data Availability

The data is available from the corresponding author on reasonable requests.

## References

[1] Palareti G (2014). How I treat isolated distal deep vein thrombosis (IDDVT). Blood.

[2] Schellong SM, Goldhaber SZ, Weitz JI, Ageno W, Bounameaux H, Turpie AGG (2019). Isolated Distal Deep Vein Thrombosis: Perspectives from the GARFIELD-VTE Registry. Thromb Haemost.

[3] Elmi G, Rinaldi ER, Domanico A, Aluigi L (2020). Calf deep vein thrombosis – clinical relevance, diagnostic approaches and therapeutic options. Vasa.

[4] Franco L, Giustozzi M, Agnelli G, Becattini C (2017). Anticoagulation in patients with isolated distal deep vein thrombosis: a meta-analysis. J Thromb Haemost.

[5] Henry JC, Satiani B (2014). Calf Muscle Venous Thrombosis: A Review of the Clinical Implications and Therapy. Vasc Endovascular Surg.

[6] Caggiati A, Bergan JJ, Gloviczki P, Jantet G, Wendell-Smith CP, Partsch H (2002). Nomenclature of the veins of the lower limbs: An international interdisciplinary consensus statement. J Vasc Surg.

[7] Kakkos SK, Gohel M, Baekgaard N, Bauersachs R, Bellmunt-Montoya S, Black SA (2021). Editor’s Choice – European Society for Vascular Surgery (ESVS) 2021 Clinical Practice Guidelines on the Management of Venous Thrombosis. Eur J Vasc Endovasc Surg.

[8] Stevens SM, Woller SC, Kreuziger LB, Bounameaux H, Doerschug K, Geersing G-J (2021). Antithrombotic Therapy for VTE Disease. Chest.

[9] Mazzolai L, Ageno W, Alatri A, Bauersachs R, Becattini C, Brodmann M (2022). Second consensus document on diagnosis and management of acute deep vein thrombosis: updated document elaborated by the ESC Working Group on aorta and peripheral vascular diseases and the ESC Working Group on pulmonary circulation and right ventricular function. Eur J Prev Cardiol.

[10] Sales CM, Haq F, Bustami R, Sun F (2010). Management of isolated soleal and gastrocnemius vein thrombosis. J Vasc Surg.

[11] Lautz TB, Abbas F, Walsh SJN, Chow C, Amaranto DJ, Wang E (2010). Isolated Gastrocnemius and Soleal Vein Thrombosis: Should These Patients Receive Therapeutic Anticoagulation?. Ann Surg.

[12] Kret MR, Liem TK, Mitchell EL, Landry GJ, Moneta GL (2013). Isolated calf muscular vein thrombosis is associated with pulmonary embolism and a high incidence of additional ipsilateral and contralateral deep venous thrombosis. J Vasc Surg Venous Lymphat Disord.

[13] MacDonald PS, Kahn SR, Miller N, Obrand D (2003). Short-term natural history of isolated gastrocnemius and soleal vein thrombosis. J Vasc Surg.

[14] Schwarz T, Schmidt B, Beyer J, Schellong SM (2001). Therapy of isolated calf muscle vein thrombosis with low-molecular-weight heparin. Blood Coag Fibrinol.

[15] Schwarz T, Buschmann L, Beyer J, Halbritter K, Rastan A, Schellong S (2010). Therapy of isolated calf muscle vein thrombosis: A randomized, controlled study. J Vasc Surg.

[16] Barco S, Corti M, Trinchero A, Picchi C, Ambaglio C, Konstantinides SV (2017). Survival and recurrent venous thromboembolism in patients with first proximal or isolated distal deep vein thrombosis and no pulmonary embolism. J Thromb Haemost.

[17] Galanaud J-P, Sevestre-Pietri M-A, Bosson J-L, Laroche J-P, Righini M, Brisot D (2009). Comparative study on risk factors and early outcome of symptomatic distal versus proximal deep vein thrombosis: Results from the OPTIMEV study. Thromb Haemost.

[18] Schellong S, Ageno W, Casella IB, Chee KH, Schulman S, Singer DE (2022). Profile of Patients with Isolated Distal Deep Vein Thrombosis versus Proximal Deep Vein Thrombosis or Pulmonary Embolism: RE-COVERY DVT/PE Study. Semin Thromb Hemost.

[19] Page MJ, McKenzie JE, Bossuyt PM, Boutron I, Hoffmann TC, Mulrow CD, The PRISMA (2020). statement: an updated guideline for reporting systematic reviews. BMJ.

[20] Righini M, Galanaud J-P, Guenneguez H, Brisot D, Diard A, Faisse P (2016). Anticoagulant therapy for symptomatic calf deep vein thrombosis (CACTUS): a randomised, double-blind, placebo-controlled trial. The Lancet Haematology.

[21] Guyatt G, Oxman AD, Akl EA, Kunz R, Vist G, Brozek J (2011). GRADE guidelines: 1. Introduction—GRADE evidence profiles and summary of findings tables. J Clin Epidemiol.

[22] DerSimonian R, Laird N (1986). Meta-analysis in clinical trials. Control Clin Trials.

[23] Higgins JPT (2003). Measuring inconsistency in meta-analyses. BMJ.

[24] Ageno W, Bertù L, Bucherini E, Camporese G, Dentali F, Iotti M, et al. Rivaroxaban treatment for six weeks versus three months in patients with symptomatic isolated distal deep vein thrombosis: randomised controlled trial. BMJ 2022:e072623. 10.1136/bmj-2022-072623. 10.1136/bmj-2022-072623 PMC9682494 36520715

[25] Sartori M, Migliaccio L, Favaretto E, Palareti G, Cosmi B (2014). Two years outcome of isolated distal deep vein thrombosis. Thromb Res.

[26] Galanaud J-P, Sevestre M-A, Genty C, Kahn SR, Pernod G, Rolland C (2014). Incidence and predictors of venous thromboembolism recurrence after a first isolated distal deep vein thrombosis. J Thromb Haemost.

[27] Jørgensen CT, Tavoly M, Førsund E, Pettersen HH, Tjønnfjord E, Ghanima W, et al. Incidence of bleeding and recurrence in isolated distal deep vein thrombosis: findings from the Venous Thrombosis Registry in Østfold Hospital. Journal of Thrombosis and Haemostasis 2023:S1538783623005147. 10.1016/j.jtha.2023.06.028. 10.1016/j.jtha.2023.06.028 37394122

[28] Sterne JAC, Sutton AJ, Ioannidis JPA, Terrin N, Jones DR, Lau J (2011). Recommendations for examining and interpreting funnel plot asymmetry in meta-analyses of randomised controlled trials. BMJ.

[29] Ho P, Lim HY, Chua CC, Sleeman M, Tacey M, Donnan G (2016). Retrospective review on isolated distal deep vein thrombosis (IDDVT) — A benign entity or not?. Thromb Res.

[30] Utter GH, Dhillon TS, Salcedo ES, Shouldice DJ, Reynolds CL, Humphries MD (2016). Therapeutic Anticoagulation for Isolated Calf Deep Vein Thrombosis. JAMA Surg.

[31] Kuczmik W, Wysokinski WE, Hesley GK, Vlazny DT, Houghton DE, Swanson KE, et al. Calf Vein Thrombosis comparison of outcomes for axial and muscular venous thrombosis. Thromb Haemost. 2021;121:216–23. 10.1055/s-0040-1715646. 10.1055/s-0040-1715646 32828073

[32] Horner D, Hogg K, Body R, Nash MJ, Baglin T, Mackway-Jones K (2014). The Anticoagulation of Calf Thrombosis (ACT) Project. Chest.

