# Supplementary figures and images for: Recurrence in isolated distal DVT after anticoagulation: a systematic review and meta-analysis of axial and muscular venous thrombosis
Source: Thromb J. 2024 Jul 1;22:57. doi: 10.1186/s12959-024-00623-6 (PMC11218106; doi:10.1186/s12959-024-00623-6)

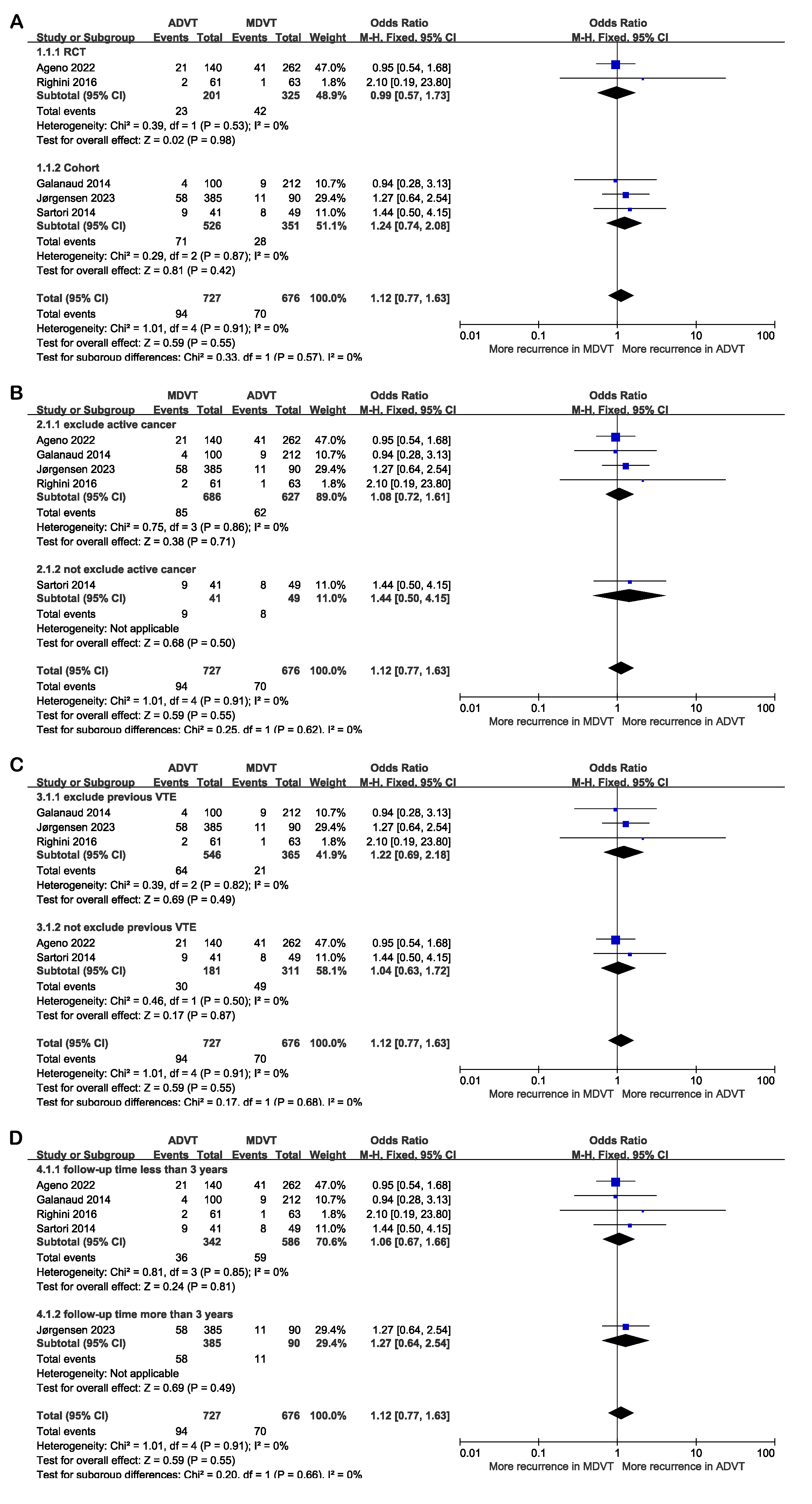

Supplement: Supplementary file 1 — Supplementary Material 1. [file 12959_2024_623_MOESM1_ESM.jpg]
