# Supplementary material for: Recurrence in isolated distal DVT after anticoagulation: a systematic review and meta-analysis of axial and muscular venous thrombosis
Source: Thromb J. 2024 Jul 1;22:57. doi: 10.1186/s12959-024-00623-6 (PMC11218106; doi:10.1186/s12959-024-00623-6)
Supplement: Supplementary file 2 — Supplementary Material 2. [file 12959_2024_623_MOESM2_ESM.docx]

**Search strategy**

**Pubmed**

(“calf”[All Fields] OR “calves”[All Fields] OR "crural"[All Fields] OR "lower leg"[All Fields] OR “lower limb” [All Fields] OR” lower extremity” [All Fields] OR "below knee"[All Fields] OR "distal"[All Fields] OR "axial"[All Fields] OR "peroneal"[All Fields] OR "fibular"[All Fields] OR "tibial"[All Fields] OR "muscular"[All Fields] OR "gastrocnemial"[All Fields] OR "soleal"[All Fields]) AND (vein [MeSH Terms] OR veins[All Fields] OR venous [All Fields]) AND (“thrombosis”[MeSH Terms] OR “phlebothrombosis” [All Fields] OR “DVT”[All Fields] OR “IDDVT”[All Fields]) AND (“pulmonary embolism”[MeSH Terms] OR “pulmonary thromboembolism”[All Fields] OR “PE”[All Fields] OR “propagation”[All Fields] OR “extension”[All Fields] OR “progression”[All Fields] OR “spreading”[All Fields] OR “advancement”[All Fields] OR “recurrence”[MeSH Terms] OR “recrudescence”[All Fields] OR “relapse”[All Fields])

**Embase**

#1 'calf':ab OR 'calves':ab OR 'crural':ab OR 'lower leg':ab OR 'lower limb':ab OR 'lower extremity':ab OR 'below knee':ab OR 'distal':ab OR 'axial':ab OR 'peroneal':ab OR 'fibular':ab OR 'tibial':ab OR 'muscular':ab OR 'gastrocnemial':ab OR 'soleal':ab

#2 'vein'/exp OR ‘veins’:ab OR ‘venous’:ab

#3 'thrombosis'/exp OR 'phlebothrombosis':ab OR 'dvt':ab OR 'iddvt':ab

#4 'pulmonary embolism'/exp OR 'pulmonary thromboembolism':ab OR 'pe':ab OR 'propagation':ab OR 'extension':ab OR 'progression':ab OR 'spreading':ab OR 'advancement':ab OR 'recurrence'/exp OR 'recrudescence':ab OR 'relapse':ab OR 'hemorrhage'/exp OR 'bleeding':ab

#5 #1AND #2 AND #3 AND #4

**Cochrane Library**

#1 'calf' OR 'calves' OR 'crural' OR 'lower leg' OR 'lower limb'OR 'lower extremity' OR 'below knee' OR 'distal' OR 'axial' OR 'peroneal' OR 'fibular' OR 'tibial' OR 'muscular' OR 'gastrocnemial' OR 'soleal'

#2 'vein' OR ‘veins’ OR ‘venous’

#3 'thrombosis' OR 'phlebothrombosis' OR 'dvt' OR 'iddvt'

#4 'pulmonary embolism' OR 'pulmonary thromboembolism' OR 'pe' OR 'propagation' OR 'extension' OR 'progression'OR 'spreading' OR 'advancement' OR 'recurrence' OR 'recrudescence' OR 'relapse'

#5 #1 AND #2 AND #3 AND #4

**Web of science**

(ALL=(calf) OR ALL=(calves) OR ALL=(crural) OR ALL=(lower leg) OR ALL=(lower limb) OR ALL=(lower extremity) OR ALL=(below knee) OR ALL=(distal) OR ALL=(axial) OR ALL=(peroneal) OR ALL=(fibular) OR ALL=(tibial) OR ALL=(muscular) OR ALL=(gastrocnemial) OR ALL=(soleal)) AND (ALL=(vein) OR ALL=(veins) OR ALL=(venous)) AND (ALL=(thrombosis) OR ALL=(phlebothrombosis) OR ALL=(dvt) OR ALL=(iddvt)) AND (ALL=(pulmonary embolism) OR ALL=(pulmonary thromboembolism) OR ALL=(pe) OR ALL=(propagation) OR ALL=(extension) OR ALL=(progression) OR ALL=(spreading) OR ALL=(advancement) OR ALL=(recurrence) OR ALL=(recrudescence) OR ALL=(relapse))
