# Supplementary material for: Recurrence in isolated distal DVT after anticoagulation: a systematic review and meta-analysis of axial and muscular venous thrombosis
Source: Thromb J. 2024 Jul 1;22:57. doi: 10.1186/s12959-024-00623-6 (PMC11218106; doi:10.1186/s12959-024-00623-6)
Supplement: Supplementary file 3 — Supplementary Material 3. [file 12959_2024_623_MOESM3_ESM.docx]

**Supplementary Material**

**Table 1. Quality assessment of the two RCTs**

**Table 2. Quality assessment of the four non-randomized prospective trials**

| **Study ID** | **Selection** | | | | **Comparability** | **Outcome** | | |
| --- | --- | --- | --- | --- | --- | --- | --- | --- |
|  | **Representativeness of the exposure** | **Selection of the non-exposure** | **Ascertainment of the exposure (IDDVT location)** | **No outcome was present at start of study** | **Comparability of ADVT and MDVT** | **Assessment of the outcome (recurrence)** | **follow-up long enough** | **Adequacy of follow up** |
| Jørgensen 2023 | * | * | * | * | * | * | * | * |
| Galanaud 2014 | * | * | * | * | * | * | * | * |
| Sartori 2014 | * | * | * | * | * | * | * | * |
